# Outcome of older (≥70 years) APL patients frontline treated with or without arsenic trioxide—an International Collaborative Study

**DOI:** 10.1038/s41375-020-0758-4

**Published:** 2020-02-19

**Authors:** Sabine Kayser, Ramy Rahmé, David Martínez-Cuadrón, Gabriel Ghiaur, Xavier Thomas, Marta Sobas, Agnes Guerci-Bresler, Ana Garrido, Arnaud Pigneux, Cristina Gil, Emmanuel Raffoux, Mar Tormo, Norbert Vey, Javier de la Serna, Olga Salamero, Eva Lengfelder, Mark J. Levis, Pierre Fenaux, Miguel A. Sanz, Uwe Platzbecker, Richard F. Schlenk, Lionel Adès, Pau Montesinos

**Affiliations:** 1 Medical Clinic and Policlinic I, Hematology and Cellular Therapy, University Hospital Leipzig, Leipzig, Germany; 2 German Cancer Research Center (DKFZ), Heidelberg, Germany; 3 Department of Internal Medicine V, Heidelberg University Hospital, Heidelberg, Germany; 4 Hôpital Saint Louis, Université Paris Diderot, Paris, France; 5 Hematology Department, Hospital Universitari i Politècnic, La Fe, Avinguda Fernando Abril Martorell, 106, 46026 València, Spain; 6 CIBERONC, Instituto Carlos III, Madrid, Spain; 7 Sidney Kimmel Comprehensive Cancer Center, Johns Hopkins University, Baltimore, MD USA; 8 Hospices Civils de Lyon, Centre Hospitalier Lyon-Sud, Pierre Bénite, Lyon, France; 9 Department of Hematology, Blood Neoplasms and Bone Marrow Transplantation, Wroclaw Medical University, Wroclaw, Poland; 10 Department of Hematology, Nancy University Hospital, Nancy, France; 11 Hospital de la Santa Creu i Sant Pau, Universitat Autònoma de Barcelona, Barcelona, Spain; 12 Department of Hematology, Bordeaux University Hospital, Bordeaux, France; 13 Hospital General, Alicante, Spain; 14 Hôpital Saint Louis, Université Paris Diderot, Paris, France; 15 Hematology Department, Hospital Clínico Universitario, INCLIVA Research Institute, University of Valencia, Valencia, Spain; 16 Institut Paoli Calmette, Marseille, France; 17 Hospital 12 de Octubre, Madrid, Spain; 18 Hospital Universitario Vall d´Hebron, Barcelona, Spain; 19 Department of Hematology and Oncology, University Hospital Mannheim, Mannheim, Germany; 20 NCT Trial Center, National Center for Tumor Diseases, German Cancer Research Center and Heidelberg University Hospital, Heidelberg, Germany

**Keywords:** Acute myeloid leukaemia, Clinical genetics

## Abstract

Data on outcome in older (≥70 years) patients with acute promyelocytic leukemia after treatment with arsenic trioxide (ATO) compared with standard chemotherapy (CTX) is scarce. We evaluated 433 patients (median age, 73.4 years) treated either with ATO+ all-trans retinoic acid (ATO/ATRA; *n* = 26), CTX/ATRA + ATO during consolidation (CTX/ATRA/ATO; *n* = 148), or with CTX/ATRA (*n* = 259). Median follow-up for overall survival (OS) was 4.8 years. Complete remissions (CR) were achieved in 92% with ATO/ATRA and 82% with CTX/ATRA; induction death rates were 8% and 18%, respectively. For analysis of postremission outcomes we combined the ATO/ATRA and CTX/ATRA/ATO groups (ATO/ATRA ± CTX). Cumulative incidence of relapse (CIR) was significantly lower after ATO/ATRA ± CTX compared with CTX/ATRA (*P* < 0.001). The same held true when restricting the analysis according to the treatment period after the year 2000. OS of patients in CR1 was not different between ATO/ATRA ± CTX compared with CTX/ATRA (*P* = 0.20). High (>10 × 10^9^/l) white blood cell (WBC) counts at diagnosis were associated with higher CIR (*P* < 0.001) compared with lower WBC in the CTX/ATRA group, but not in the ATO/ATRA ± CTX group (*P* = 0.48). ATO, when added to ATRA or CTX/ATRA is feasible and effective in elderly patients for remission induction and consolidation, particularly in patients with high WBC at diagnosis.

## Introduction

Upfront arsenic trioxide (ATO) and all-trans retinoic acid (ATRA) combination has been proven to be highly effective in acute promyelocytic leukemia (APL) and has become standard therapy in younger adult, nonhigh-risk patients [1, 2]. Within the pre-ATO era, high white blood cell (WBC) counts were identified as a risk factor for relapse [3–6]. After initial induction treatment with ATRA and idarubicin (AIDA), subsequent risk-adapted consolidation therapy has shown to partly equalize the risk of relapse between APL-risk groups [5, 7].

To date, reports on outcome of older APL patients (≥70 years) with ATO/ATRA are scarce [8, 9]. Although the approval of ATO in the USA and Europe was based on the randomized APL0406 study in patients 18–70 years of age [1, 2] no upper age limit for the treatment with ATO/ATRA was included in both labels. Thus, clinical data on patients above the age of 70 years treated with different regimens will help clinicians to identify best treatment options for their older APL patients.

In addition, a high mortality before start and during the first day of treatment is still a major issue in APL, particularly in older patients according to the Swedish adult acute leukemia registry [10]. Early death rates after ATRA± anthracycline-based induction therapy were as high as 60% in patients above the age of 80 years and still 18.8% in patients aged 50–59 years [10]. Furthermore, despite dose reduction of chemotherapy (CTX) in older patients [11–13] non-relapse mortality during postremission therapy remained high, with 10–18.6% dying mainly due to infections, whereas it was much lower (6.9%) after therapy with ATO [8]. In contrast to CTX/ATRA, only single cases with a secondary malignancy are reported after ATO treatment [8].

The objectives of our study were to evaluate characteristics and outcome of older (≥70 years) APL patients treated with different treatment strategies in a large, international cooperative cohort of patients.

## Patients and methods

### Patients and treatment

Data on 475 patients aged ≥70 years, reported either to the multicenter, multinational registries German Intergroup Napoleon registry (*n* = 28), Spanish PETHEMA (Programa Español de Tratamientos en Hematología, *n* = 211), or French APL Group (*n* = 228) or to the local data base at the Johns Hopkins University of Maryland, USA (*n* = 8), were collected in a large international collaboration. All patients were treated between 1990 and 2018. Patients who were treated less intensively with single-agent ATRA or in combination with low-dose cytarabine or comparable CTX (*n* = 38) or patients with lacking data on response and survival (*n* = 4) were excluded from analysis. The final study cohort consisted of *n* = 433 patients. Diagnosis of APL was based on genetic analysis as well as on French–American–British Cooperative Group criteria [14], and, after 2003, on revised International Working Group criteria [15]. Chromosome banding was performed using standard techniques, and karyotypes were described according to the International System for Human Cytogenetic Nomenclature [16]. The diagnosis was confirmed by either reverse-transcriptase polymerase chain reaction (RT-qPCR) or fluorescence in situ hybridization detection by standard methods. *FLT3* mutation screening for internal tandem duplications (ITD) and point mutations within the tyrosine kinase domain (TKD) was carried out as previously described [17, 18]. Data collection and analysis were approved by the local Institutional Review Boards.

### Treatment

Two-hundred and fifty-nine patients (60%) were treated with ATRA and an anthracycline (daunorubicin or idarubicin) as induction and different CTX in combination with ATRA as consolidation therapy according to treatment protocols active in various institutions and cooperative groups (Supplementary Table 1). These protocols included the PETHEMA LPA96 [19] (*n* = 23), LPA99 [20] (*n* = 45), LPA2005 [21] (*n* = 76), and LPA2012 (ClinicalTrials.gov Identifier: NCT02020161; *n* = 31), the French APL93 [7] (*n* = 34) and APL2000 [22] (*n* = 41) trials, as well as standard AIDA-based CTX [23] (*n* = 9). Twenty-six (6%) patients were treated with ATO/ATRA [1] (including one dose of idarubicin, *n* = 1 or daunorubicin for ATRA-syndrome, *n* = 1; and 5 days of decitabine, *n* = 1). In addition, 148 (34%) patients were treated with CTX/ATRA + ATO according to the French APL2006 trial [24] (*n* = 144), or according to Gore et al. [25] (*n* = 4). Response and treatment failure were assessed according to Cheson et al. [15].

### Statistical analyses

Overall survival (OS) was defined as recommended [15]. Induction death was defined as death occurring before complete remissions (CR) evaluation, occurring at the latest within 43 days after treatment initiation in our cohort.

Comparisons of patient characteristics were performed with the Kruskal–Wallis rank sum test for continuous variables and Fisher’s exact test for categorical variables, respectively. The median follow-up time was computed using the reverse Kaplan–Meier estimate [26]. The Kaplan–Meier method was used to estimate the distribution of OS [27]. Confidence interval (CI) estimation for survival curves was based on the cumulative hazard function using Greenwood’s formula for variance estimation. Logrank tests were employed to compare survival curves between groups. For cumulative incidence of relapse (CIR) and death (CID), equality of cumulative incidences between the treatment groups was evaluated using the Gray’s test [28]. A cause-specific Cox proportional hazards regression model was used to identify prognostic variables for relapse [29]. The following variables were included in the Cox model: age at diagnosis, gender, APL treatment (ATO/ATRA ± CTX vs. CTX/ATRA), and WBC count (dichotomized, ≤10 × 10^9^/l vs. >10 × 10^9^/l). All statistical analyses were performed with the statistical software environment R, version 3.3.1, using the R packages, and survival, version 2.39-5 [30].

## Results

In total, 433 older APL patients, diagnosed between 1990 and 2015 from four study groups/institutions in the USA and Europe, were included. Median age was 73.4 years (range, 70–89 years).

Risk categorization based on WBC count at diagnosis was available in 429 (99%) of the 433 patients and was low-/intermediate-risk (WBC ≤ 10 × 10^9^/l) in 339 (79%) and high-risk (WBC > 10 × 10^9^/l) in 90 (21%) patients.

Information on cytogenetics was available in 381 (88%) patients. In 26 (7%) of the 381 patients t(15;17) could only be detected by FISH and/or RT-qPCR, whereas cytogenetics showed a normal karyotype. In 238 (67%) of the remaining 355 patients, the balanced t(15;17) translocation was the sole abnormality, whereas in 117 (33%) patients, the translocation was accompanied by additional cytogenetic abnormalities, most frequently trisomy 8 (*n* = 7; 6%) or t(15;17) within a complex karyotype (≥2 cytogenetic abnormalities in addition to t(15;17); *n* = 12; 10%).

Regarding *FLT3*-ITD and TKD mutations, information was available in only 56 (13%) of the 433 patients. Of those, eight (14%) patients had *FLT3*-ITD and two (4%) had a *FLT3*-TKD mutation. Patients with *FLT3*-ITD mutations had significantly higher WBC at diagnosis as compared with *FLT3* wild type patients (*P* < 0.001).

Information on the *PML-RARA* transcript isoform (breakpoint cluster region (BCR)) was available in 292 patients; 125 (43%) had the short isoform (BCR3). Baseline characteristics according to the frontline strategy are shown in Table 1.

**Table 1:** Comparison of presenting clinical and laboratory findings according to the applied therapy of older patients with acute promyelocytic leukemia.

Characteristics	CTX/ATRA *n* = 259	CTX/ATRA/ ATO *n* = 148	ATO/ATRA *n* = 26	*P* value
Age, years				
Median	73.4	73.2	76	0.07
Range	70–88.7	70–89	71–87	
Gender, no. (%)				
Male	123 (47)	80 (54)	18 (69)	0.07
Female	136 (53)	68 (46)	8 (31)	
WBC, ×10^9^/l				
Median	2.1	1.3	0.8	<0.001
Range	0.1–764	0.2–136	0.2–3.6	
Missing	4	0	0	
Hemoglobin, g/dl				
Median	9.2	9.7	9.2	0.20
Range	4.0–15.2	4.6–14.3	6.0–14.0	
Missing	14	2	17	
Platelet count, ×10^9^/l				
Median	34	41	43	0.08
Range	3–213	4–261	11–195	
Missing	8	3	2	
Percentage of PB blasts^a^				
Median	61	6	1	<0.001
Range	0–100	0–99	0–67	
Missing	53	15	7	
Percentage of BM blasts^a^				
Median	80	74	50	0.005
Range	13–100	12–98	3–90	
Missing	42	0	11	
High-risk^b^	72 (28)	18 (12)	0	<0.001
Missing	4	0	0	

Percentages may not add to 100 because of rounding.

*ATO* arsenic trioxide, *ATRA* all-trans retinoic acid, *BM* bone marrow, *CTX* chemotherapy, *PB* peripheral blood, *WBC* white blood cell counts.

^a^Blast cells included malignant promyelocytes.

^b^High risk: WBC > 10 × 10^9^/l.

Median age was in trend higher in the ATO/ATRA group as compared with the CTX/ATRA and CTX/ATRA/ATO groups. Median WBC, percentage of peripheral blood and bone marrow blasts as well as risk categorization were significantly lower in the ATO/ATRA ± CTX group as compared with the CTX/ATRA group.

### Response to induction therapy

Overall, 356 patients achieved a CR (82%), two patients had refractory disease (0.5%) and 75 patients died during induction therapy (17%). The rate of death during induction therapy was lower in patients receiving ATO/ATRA (*n* = 2/26; 8%) as compared with those receiving CTX/ATRA (*n* = 73/407; 18%) as induction therapy (*P* = 0.28). The rate of deaths during induction therapy decreased over time from 28% (17 of 60 patients) in 1990–1999, to 15% (29 of 188 patients) in 2000–2009 and 16% (29 of 185 patients) in 2010–2018 (*P* = 0.07). Main causes of deaths were bleeding/hemorrhage (*n* = 16), infections (*n* = 17), differentiation syndrome (*n* = 9), multi-organ failure (*n* = 3), as well as other causes (not further specified; *n* = 30).

CRs were achieved after induction therapy in 82% with CTX/ATRA (*n* = 332/407) and in 92% with ATO/ATRA (*n* = 24/26; Table 2; *P* = 0.29). In a logistic regression model on response to induction therapy WBC count above 10 × 10^9^/l (*P* < 0.001) and age above 75 years (*P* = 0.04) were unfavorable factors, whereas treatment with ATO/ATRA was a beneficial, but not a significant factor (*P* = 0.34; Table 3). APL was refractory after CTX/ATRA treatment in two patients, who died after 51 and 477 days from diagnosis, respectively. None of the patients in the ATO/ATRA group was refractory.

**Table 2:** Response to induction therapy according to treatment strategy.

% (*N*)	CTX/ATRA *N* = 407	ATO/ATRA *N* = 26
CR	82 (332)	92 (24)
RD	0.5 (2)	–
ID	18 (73)	8 (2)

Missing data, *n* = 3 (CTX/ATRA). Percentages may not add to 100 because of rounding.

*ATO* arsenic trioxide, *ATRA* all-trans retinoic acid, *CR* complete remission, *CTX* chemotherapy, *ID* induction death, *N* numbers, *RD* resistant disease.

**Table 3:** Logistic regression model on response to induction therapy.

	Regression model on response to induction therapy	
	OR	*P* value
Age above 75 years	0.55	0.030
WBC (>10 × 10^9^/l)	0.26	<0.001
ATO/ATRA	2.21	0.30
Male gender	0.72	0.22

*ATO* arsenic trioxide, *ATRA* all-trans retinoic acid, *CTX* chemotherapy, *OR* odds ratio, *WBC* white blood cell count.

Of twelve patients with a complex karyotype, eight were treated with AIDA-based regimen, whereas four patients received ATO/ATRA. Two patients treated with AIDA died after induction therapy and two patients relapsed, whereas no induction death or relapse occurred after ATO/ATRA treatment.

### Survival analysis

**Fig. 1:**
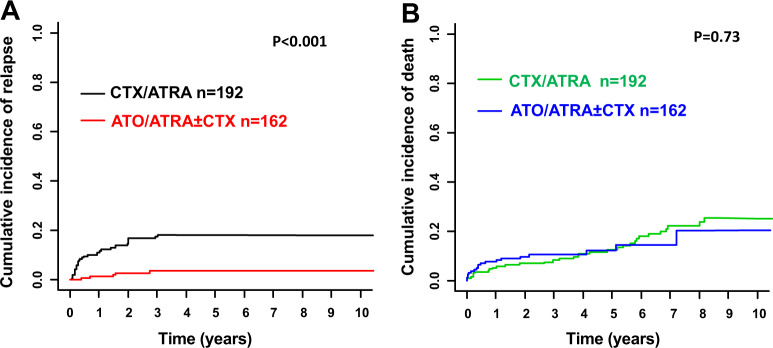
Cumulative incidence of relapse (CIR, **a**) and cumulative incidence of death (CID, **b**) according to treatment strategy. *ATO* arsenic trioxide, *ATRA* all-trans retinoic acid, *CTX* chemotherapy, *n* number.

**Fig. 2:**
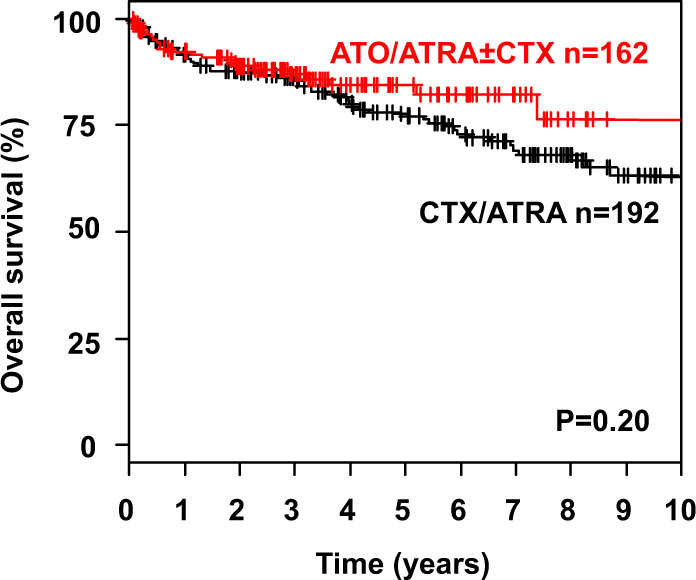
Overall survival. Overall survival of patients achieving a complete remission after induction therapy according to treatment strategy.

**Fig. 3:**
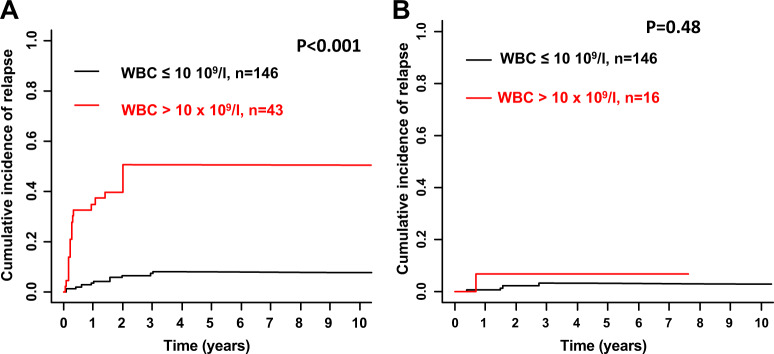
Cumulative incidence of relapse according to risk category and treatment. **a** Cumulative incidence of relapse according to risk category (white blood cell count ≤10 × 10^9^/l vs. >10 × 10^9^/l) in patients treated with all-trans retinoic acid and chemotherapy. **b** Cumulative incidence of relapse according to risk category (white blood cell count ≤10 × 10^9^/l vs. >10 × 10^9^/l) in patients treated with arsenic trioxide-based regimens.

Median follow-up for survival was 4.8 years (95% CI, 4.3–5.6 years). Overall, the estimated 5-year OS rate was 66% (95% CI, 61–71%). As we were interested in the effect of ATO on CIR and CID, as well as survival of CR patients we combined ATO/ATRA and CTX/ATRA + ATO in one group (ATO/ATRA ± CTX). Treatment with ATO/ATRA ± CTX was associated with significantly reduced CIR after 5 years (4% vs. 18%; *P* < 0.001; Fig. 1a) with no significant differences in CID (12% and 13%; *P* = 0.73; Fig. 1b) and OS rates of CR patients (84% (95% CI, 78–91) vs. 77% (95% CI, 71–84%); *P* = 0.20; Fig. 2) as compared with CTX/ATRA. When restricting the analysis according to the treatment period after the year 2000, ATO/ATRA ± CTX was still associated with significantly lower CIR (*P* = 0.02) as compared with CTX/ATRA. Of note, higher WBC counts at diagnosis (>10 × 10^9^/l) were associated with an increased CIR in CTX/ATRA (*P* < 0.001; Fig. 3a), but not in ATO/ATRA ± CTX (*P* = 0.48; Fig. 3b).

Overall, five patients relapsed after ATO/ATRA ± CTX (median time from CR to relapse, 18.2 months) as compared with 33 after CTX/ATRA (median time from CR to relapse, 5 months). Of those, risk categorization was high in one patient of the ATO/ATRA ± CTX group as compared with 22 patients of the CTX/ATRA group.

Only two patients experienced besides a hematological a CNS relapse. Both relapses occurred after AIDA-based therapy. Radiotherapy was applied in one patient, whereas the other patient received no treatment. Both patients died shortly after relapse (13 days and 98 days).

Of the CTX/ATRA group eight patients received ATO and additionally five patients ATO/ATRA as salvage therapy. Overall, 12 of the 13 relapsed patients of the CTX/ATRA group achieved a second remission, whereas one patient died during salvage therapy. Multivariate analysis showed that higher WBC count (>10 × 10^9^/l; hazard ratio (HR), 7.90; *P* < 0.001) had an adverse impact on relapse, whereas therapy with ATO/ATRA ± CTX (HR, 0.23; *P* = 0.003) was beneficial (Table 4).

**Table 4:** Cause-specific Cox model on relapse risk.

	Cause-specific Cox model on relapse risk	
	HR	*P* value
Age above 75 years	1.07	0.86
WBC (>10 × 10^9^/l)	7.90	<0.001
ATO/ATRA ± CTX	0.23	0.003
Male gender	0.99	0.99

*ATO* arsenic trioxide, *ATRA* all-trans retinoic acid, *CTX* chemotherapy, *HR* hazard ratio, *OR* odds ratio, *WBC* white blood cell count.

Fifty-five patients died in remission (CTX/ATRA, *n* = 37; ATO/ATRA ± CTX, *n* = 18), mainly due to infections (*n* = 12; CTX/ATRA, *n* = 7 and ATO/ATRA + CTX, *n* = 5), bleeding/hemorrhage (*n* = 9; CTX/ATRA, *n* = 2 and ATO/ATRA + CTX, *n* = 7), cardiac arrest + aneurysm rupture in the brain (ATO/ATRA + CTX, *n* = 1), encephalitis/cerebellitis (ATO/ATRA + CTX, *n* = 2), prostate cancer (ATO/ATRA + CTX, *n* = 1), and unknown causes (*n* = 30; CTX/ATRA, *n* = 28 and ATO/ATRA + CTX, *n* = 2).

Among patients achieving CR after intensive therapy, the median follow-up time was 3.4 years for ATO/ATRA ± CTX and 7.2 years for CTX/ATRA. Data on the development of secondary neoplasms were available in 163 patients. Of those, 11 (7%) patients developed a secondary neoplasm (gastro-intestinal cancer, *n* = 4; lung cancer, *n* = 3; neuroendocrine carcinoma, *n* = 1; and secondary acute myeloid leukemia, *n* = 3) after a median of 61.5 months (range, 5.5–85 months) from the time point of CR. All of them had been treated with CTX/ATRA. In a competing risk analysis among patients in CR after intensive therapy, the rate of secondary malignancies was significantly lower after ATO/ATRA ± CTX as compared with CTX/ATRA (*P* = 0.02).

## Discussion

Our retrospective analysis of a large cohort of 433 older APL patients, spanning a time period of almost 28 years, shows a higher response rate after treatment with ATO/ATRA as compared with CTX/ATRA, primarily due to a lower rate of induction deaths, which is in line with published data in younger APL patients [1, 9]. The rate of induction deaths decreased from 28% between 1990 and 1999 to 15% and 16% of the following decades, which may be attributable to improved supportive care and awareness of APL as a medical emergency. This finding suggests that the clear guidance in international recommendations about early supportive care has had an effect in recent years [31]. Thus, an effective strategy to overcome induction mortality is due to standardized guidelines along with consultative support and sharing of expertise [32]. Main causes of induction death after CTX/ATRA included bleeding/hemorrhage and infections, which is in line with previously published data [10, 33, 34]. However, 30 patients experienced induction death due to other causes, which might simply be related to the fact, that older patients might have more frequent and severe comorbidities and thus lower capacity to tolerate CTX [10]. The main limitation of CTX/ATRA treatment is the high rate of induction death, whereas treatment with ATO/ATRA seems to be safer and being associated with a low-induction death rate. Thus, treating patients with ATO/ATRA during induction seems to be rational rather than in consolidation, when the greatest risk has passed [35]. However, in our cohort the number of patients treated with ATO/ATRA during induction was very low and induction mortality was high after CTX/ATRA, particularly in the earlier treatment period before 1999. Therefore, a comparison of OS between the CTX/ATRA and ATO/ATRA groups is hampered. Thus, we focused in time-to-event analyses on patients in first CR.

As a potential selection bias, none of the patients treated with ATO/ATRA in induction therapy had high risk (WBC count >10 × 10^9^/l) at diagnosis as compared with 28% in the CTX/ATRA group, which is a known risk factor for bleeding diathesis [3]. In addition, as a further potentially selection bias, percentage of peripheral blood and bone marrow blasts were also unequally distributed between the CTX/ATRA and ATO-based groups.

Regarding postremission outcome, results of the randomized North American Leukemia Intergroup Study C9710 on 481 APL patients (age range, 15–79 years including 77 patients above the age of 60 years) evaluating ATO in first-line therapy during consolidation demonstrated that ATO further reduced the risk of relapse and improved survival as compared with consolidation with daunorubicin/cytarabine [35]. In contrast, a subgroup analysis of the randomized phase-III AML17 trial of the UK National Cancer Research Institute Acute Myeloid Leukaemia Working Group on 49 older (age range, ≥60–77 years) patients showed no significant difference of the 4-year OS rate after treatment with ATO/ATRA (74%; *n* = 25) as compared with the AIDA-based regimen (80%; *n* = 24) [9]. However, this was in contrast to the total study cohort with significantly better event-free and relapse-free survival after ATO/ATRA as compared with the AIDA-based regimen. In our cohort postremission outcomes were comparable after CTX/ATRA and ATO-based treatment in patients with low-/intermediate-risk, which is in contrast to previously published data in younger adults [1, 2]. Contrary to the data of the AML17 trial [9], the ATO-based treatment resulted in significant lower CIR in patients with high-risk.

There was no difference between CTX/ATRA and ATO-based regimens with regard to deaths in remission with relatively high rates of 12% and 13%, which is probably due to the advanced age of the cohort. Besides bleeding infections were the most frequent cause of death, particularly after CTX/ATRA treatment, arguing for a CTX-free approach. Other causes were most probably related to the natural death rate.

In addition, there was a significant difference in the occurrence of secondary malignancies, since none of the patients developed a secondary therapy-related myeloid neoplasm or solid tumor after ATO-based regimens as compared with 11 (7%) patients after CTX/ATRA, including four patients, who developed a secondary malignancy within 2.5 years after achievement of CR. Although the median follow-up time was shorter after ATO-based regimens as compared with CTX/ATRA, the reported latency period between APL diagnosis and the development of a secondary malignancy of 6.6 months strongly argues for a true lower incidence after ATO/ATRA ± CTX [36]. The low-relapse rate after ATO-based therapy in our cohort is in line with others [8, 35]. Moreover, 12 relapsed patients of the CTX/ATRA group were successfully salvaged with ATO-based therapy, confirming the tolerability of this treatment option in elderly patients. In contrast to the publication of Powell et al. [35] presenting a subgroup analysis in older patients, treatment with ATO/ATRA was not superior to the standard CTX/ATRA-approach in the low-/intermediate-risk in our cohort. However, a clear and significant benefit was seen in the high-risk group. Thus, our data suggest that ATO-based regimens are effective in older patients independent of risk classification.

Regarding biological characteristics, additional cytogenetic abnormalities were present in 25% of the patients, most frequently trisomy 8, which is in line with published data [37, 38]. In addition, t(15;17) could also be found within a complex karyotype, suggesting that older age might lead to higher chromosomal instability and thus higher rate of abnormalities, as has been described [39]. Although data were limited to a low number of patients, *FLT3*-ITD mutations seem to be less frequent in older (14%) as compared with all APL patients (31%) [35]. As expected, *FLT3*-ITD mutated patients had significantly higher WBC count at diagnosis as compared with *FLT3* wild type patients [37, 40–43]. To date, there are still conflicting data regarding the impact of additional chromosomal or genetic abnormalities on outcome in APL patients [37, 40–49]. In our large cohort, the presence of additional cytogenetic abnormalities had no impact on OS (*P* = 0.99), whereas *FLT3*-ITD was associated with an adverse impact on OS (*P* < 0.001). The latter issue, however, should be interpreted with precaution due to the low availability of *FLT3* mutational status in our cohort.

Regarding the distribution of risk category according to WBC count at diagnosis, published data are again contradictory [49, 50]. Sanz et al. [49] reported that older patients seem to be more likely to present with non-high-risk APL as compared with their younger counterparts (37% vs. 18%), which in part may account for the low relapse rate observed in their publication. In contrast, Lengfelder et al. reported on 31% (*n* = 28/91) of older patients with high-risk APL [50]. In our cohort, the proportion of high-risk patients in the CTX/ATRA group was comparable with the latter publication [50], whereas it was lower in the ATO/ATRA/ ± CTX group due to study inclusion criteria [24] as well as reservation to use ATO-based therapy in high-risk patients. As expected, higher WBC count above 10 × 10^9^/l was an unfavorable factor for response to induction therapy as well as CIR and CID. Of note, no difference in outcome was observed in high-risk patients after treatment with ATO/ATRA ± CTX, whereas this was not the case in patients after treatment with CTX/ATRA, suggesting that the ATO-based regimen is also highly active within the high-risk group.

In conclusion, ATO, when added to ATRA or CTX/ATRA, is a feasible and efficacious treatment and leads to good outcomes in the primary management of older APL patients. Thus, it seems to be prudent to expand this approach to older APL patients.

## Supplementary information

Supplementary Material - Treatment schedules of the different trials
