# Publisher Correction: Outcome of older (≥70 years) APL patients frontline treated with or without arsenic trioxide—an International Collaborative Study

**DOI:** 10.1038/s41375-021-01358-3

**Published:** 2021-11-16

**Authors:** Sabine Kayser, Ramy Rahmé, David Martínez-Cuadrón, Gabriel Ghiaur, Xavier Thomas, Marta Sobas, Agnes Guerci-Bresler, Ana Garrido, Arnaud Pigneux, Cristina Gil, Emmanuel Raffoux, Mar Tormo, Norbert Vey, Javier de la Serna, Olga Salamero, Eva Lengfelder, Mark J. Levis, Pierre Fenaux, Miguel A. Sanz, Uwe Platzbecker, Richard F. Schlenk, Lionel Adès, Pau Montesinos

**Affiliations:** 1 Medical Clinic and Policlinic I, Hematology and Cellular Therapy, University Hospital Leipzig, Leipzig, Germany; 2 German Cancer Research Center (DKFZ), Heidelberg, Germany; 3 Department of Internal Medicine V, Heidelberg University Hospital, Heidelberg, Germany; 4 Hôpital Saint Louis, Université Paris Diderot, Paris, France; 5 Hematology Department, Hospital Universitari i Politècnic, València, Spain; 6 CIBERONC, Instituto Carlos III, Madrid, Spain; 7 Sidney Kimmel Comprehensive Cancer Center, Johns Hopkins University, Baltimore, MD USA; 8 Hospices Civils de Lyon, Centre Hospitalier Lyon-Sud, Lyon, France; 9 Department of Hematology, Blood Neoplasms and Bone Marrow Transplantation, Wroclaw Medical University, Wroclaw, Poland; 10 Department of Hematology, Nancy University Hospital, Nancy, France; 11 Hospital de la Santa Creu i Sant Pau, Universitat Autònoma de Barcelona, Barcelona, Spain; 12 Department of Hematology, Bordeaux University Hospital, Bordeaux, France; 13 Hospital General, Alicante, Spain; 14 Hematology Department, Hospital Clínico Universitario, INCLIVA Research Institute, University of Valencia, Valencia, Spain; 15 Institut Paoli Calmette, Marseille, France; 16 Hospital 12 de Octubre, Madrid, Spain; 17 Hospital Universitario Vall d´Hebron, Barcelona, Spain; 18 Department of Hematology and Oncology, University Hospital Mannheim, Mannheim, Germany; 19 NCT Trial Center, National Center for Tumor Diseases, German Cancer Research Center and Heidelberg University Hospital, Heidelberg, Germany

**Keywords:** Acute myeloid leukaemia, Clinical genetics

Correction to: *Leukemia* 10.1038/s41375-020-0758-4

The article Outcome of older (≥70 years) APL patients frontline treated with or without arsenic trioxide—an International Collaborative Study, written by Sabine Kayser, Ramy Rahmé, David Martínez-Cuadrón, Gabriel Ghiaur, Xavier Thomas, Marta Sobas, Agnes Guerci-Bresler, Ana Garrido, Arnaud Pigneux, Cristina Gil, Emmanuel Raffoux, Mar Tormo, Norbert Vey, Javier de la Serna, Olga Salamero, Eva Lengfelder, Mark J. Levis, Pierre Fenaux, Miguel A. Sanz, Uwe Platzbecker, Richard F. Schlenk, Lionel Adès & Pau Montesinos, was originally published Online First without Open Access. After publication in volume 34, page 2333–2341, the author decided to opt for Open Choice and to make the article an Open Access publication. Therefore, the copyright of the article has been changed to © The Author(s) 2020 and the article is forthwith distributed under the terms of the Creative Commons Attribution.

