# Supplementary material for: Outcome of older (≥70 years) APL patients frontline treated with or without arsenic trioxide—an International Collaborative Study
Source: Leukemia. 2020 Feb 19;34(9):2333–41. doi: 10.1038/s41375-020-0758-4 (PMC8318880; doi:10.1038/s41375-020-0758-4)
Supplement: Supplementary file 1 — Supplementary Material - Treatment schedules of the different trials [file 41375_2020_758_MOESM1_ESM.docx]

**Supplementary Table 1: Treatment schedules of the different trials:**

**APL 93 trial (reference 7):**

- Induction: all-trans retinoic acid (ATRA) 45 mg/m^2^ + daunorubicin 90 mg/m^2^/d, days 1-3 + cytarabine 200 mg/ m^2^/d, days 1-7
- Consolidation 1: same therapy as during induction
- Consolidation 2: ATRA + daunorubicin, days 1-3 + cytarabine 1g/m^2^/12h, days 1-4
- Maintenance: ATRA + 6MP + MTX

**APL 2000 trial (reference 22):**

- Induction: ATRA 45 mg/m^2^ + daunorubicin 60 mg/m^2^/d, days 1-3 + cytarabine 200 mg/m2/d, days 1-7
- Consolidation 1: daunorubicin 60 mg/m^2^/d, days 1-3 + cytarabine 200 mg/m^2^/d, days 1-7
- Consolidation 2: daunorubicin 45 mg/m^2^/d, days 1-3 + cytarabine 1g/m^2^/12h, days 1-4
- Maintenance: ATRA + 6-mercaptopurine + methotrexate

**APL 2006 trial (reference 24):**

- **Low risk:**
- Induction: ATRA 45 mg/m^2^ until complete remission+ idarubicin 9 mg/m^2^/d, days 3, 5 and 7
- Consolidation 1: idarubicin 9 mg/m^2^/d for 3* days combined with ATO 0.1 mg/kg/d during 25 days (*dose reduction after 09/2010 to idarubicin 9mg/m² on day 1)
- Consolidation 2: ATO (same schedule as in consolidation 1) and ATRA 45 mg/ m^2^ days 1-15
- Maintenance: for 2 years with intermittent ATRA, continuous 6-mercaptopurine and methotrexate, plus 15 day ATO cycles every 3 months during the first year
- **High risk:**
- Induction: ATRA 45 mg/m^2^ + idarubicin 9 mg/m^2^/d, days 1-3 + cytarabine 120 mg/m^2^/d, days 1-7
- Consolidation 1: idarubicin 9 mg/m^2^/d, days 1-3 + ATO 0.15 mg/kg/d x 20 days + cytarabine 60mg/m2/12h, days 1-5
- Consolidation 2: ATO (same schedule as in consolidation 1) and ATRA 45 mg/ m^2^ during 15 days
- Maintenance: for 2 years with intermittent ATRA, continuous 6-mercaptopurine and methotrexate, plus 15 day ATO cycles every 3 months during the first year

**LPA96 trial (reference 19):**

- Induction: idarubicin 12 mg/m^2^ d 2, 4, 6, 8 and ATRA 45 mg/m2/d
- Consolidation 1: idarubicin 5 mg/m^2^/d × 4
- Consolidation 2: mitoxantrone 10 mg/m^2^/d × 5
- Consolidation 3: idarubicin 12 mg/m^2^/d ×1
- Maintenance: alternant ATRA and low-dose methotrexate and 6-mercaptopurine for 2 years

**LPA99 trial (reference 20):**

- Induction: Idarubicin 12 mg/m^2^ d 2, 4, 6 + ATRA 45 mg/m2/d
- Consolidation:
  - Low-risk:
    - Idarubicin 5 mg/m^2^/d x 4
    - Mitoxantrone 10 mg/m^2^/d x 5
    - Idarubicin 12 mg/m^2^/d x 1
  - Intermediate-/ high-risk:
    - Idarubicin 7 mg/m^2^/d x 4 + ATRA 45 mg/m^2^/d x 15
    - Mitoxantrone 10 mg/m^2^/d x 5 + ATRA 45 mg/m^2^/d x 15
    - Idarubicin 12 mg/m^2^/d x 2 + ATRA 45 mg/m^2^/d x 15

**LPA2005 trial (reference 21):**

- Induction: ATRA 45 mg/m²/d + idarubicin 12 mg/m² d2, 4, 6 (for all risk-groups)
- Consolidation:
  - Low-risk:
    - Idarubicin 5 mg x4 + ATRA 45mg/m²/ days 1-15
    - Mitoxantrone 10 mg x3 + ATRA 45mg/m²/ days 1-15
    - Idarubicin 12 mg x1 + ATRA 45mg/m²/ days 1-15
  - Intermediate and high-risk:
    - Idarubicin 7 mg x4 + ATRA 45mg/m²/ days 1-15
    - Mitoxantrone 10 mg x3 + ATRA 45mg/m²/ days 1-15
    - Idarubicin 12 mg x2 + ATRA 45mg/m²/ days 1-15
- Maintenance: alternant ATRA and 6-mercaptopurine + methotrexate for 2 years (for all risk-groups)

**LPA2012 trial (ClinicalTrials.gov Identifier: NCT02020161):**

- Induction: ATRA 45mg/m²/d + idarubicin 12 mg/m² d2, 4, 6
- Consolidation:
  - IDA 5 mg x4 + ATRA 45mg/m²/ days1-15
  - MTZ 10 mg x3 + ATRA 45mg/m²/ days1-15
  - IDA 12 mg x1 + ATRA 45mg/m²/ days1-15
- Maintenance: alternant ATRA and 6-mercaptopurine + methotrexate for 2 years

**APL04/06 trial (reference 1):**

- Induction: ATO 0.15 mg/kg/day + ATRA 45mg/m²/day until complete remission
- Consolidation: ATO 0.15 mg/kg/day for 5 days per week x 4 cycles + ATRA 45 mg/m²/day for 15 days every 2 weeks x 7 cycles

**J0442 trial (reference 25):**

- Induction: ATRA 45mg/m² for 60 days + daunorubicin 60mg/m²/day, days 4,6,8
- Consolidation: cytarabine 0.667 mg/m²/day, days 1-3, daunorubicin 60mg/m²/day, days 1-3, ATO 0.15 mg/kg/day, days 1-5 (beginning on day 8 for 30 doses)
- Maintenance:
  - white blood cell count < 10x10^9^/l:
    - ATRA 45mg/m² for 15 days every 3 months for 8 cycles
  - white blood cell count > 10x10^9^/l:
    - ATRA 45mg/m² for 15 days every 3 months for 8 cycles
    - 6-mercaptopurine 60mg/m²/day + methotrexate 15mg/m²/week
